# Recurrent Versus Primary Lumbar Disc Herniation Surgery: Patient-reported Outcomes in the Swedish Spine Register Swespine

**DOI:** 10.1007/s11999-014-3596-8

**Published:** 2014-04-08

**Authors:** Peter Fritzell, Björn Knutsson, Bengt Sanden, Björn Strömqvist, Olle Hägg

**Affiliations:** 1 Department of Orthopaedic Surgery, Future Academy, Ryhov Hospital, 551 85 Jönköping, Sweden; 2 Department of Orthopaedics, Sundsvall Hospital, Sundsvall, Sweden; 3 Department of Orthopaedics, University Hospital Uppsala, Uppsala, Sweden; 4 Department of Orthopaedics, Skane University Hospital, Malmö, Sweden; 5 Spine Center Gothenburg, Gothenburg, Sweden

## Abstract

**Background:**

Lumbar disc herniation (LDH) is a common indication for lumbar spine surgery. The proportion of patients having a second surgery within 2 years varies in the literature between 0.5% and 24%, with recurrent herniation being the most common cause. Several studies have not found any relevant outcome differences between patients undergoing surgery for primary LDH and patients undergoing reoperation for a recurrent LDH, but these studies have limitations, including small sample size and retrospective design.

**Questions/purposes:**

We (1) compared patient-reported outcomes between patients operated on for primary LDH and patients reoperated on for recurrent LDH within 1 year after index surgery and (2) determined risk factors for worse outcomes.

**Methods:**

We obtained data from the Swedish National Spine Register, Swespine, where patient-reported outcomes are collected using mailed protocols at 1, 2, 5, and 10 years after surgery. Of the 13,562 patients identified who underwent LDH between January 2000 and May 2011, 13,305 (98%) underwent primary surgery for LDH and 257 (2%) underwent reoperation for a recurrent LDH within the first year. Patient-reported outcomes at 1 to 2 years were available for 8497 patients (63%), 8350 of 13,305 (63%) in the primary LDH group and 147 of 257 (57%) in the recurrent LDH group (p = 0.068). We compared leg and back pain (VAS: 0–100), function (Oswestry Disability Index [ODI]: 0–100), quality of life (EQ-5D: −0.59 to 1.0), patient satisfaction, and global assessment of leg pain between groups. We also analyzed rsik factors for worse global assessment and satisfaction.

**Results:**

Mean (95% CI) differences in improvement between groups favoring patients with primary LDH were VAS leg pain 9 (4–14), ODI 6 (3–9), and EQ-5D 0.09 (0.04–0.15). While statistically significant, these effect sizes may be lower than the minimal clinically important differences often referred to. Percentage of satisfied patients was 79% and 58% in the primary and recurrent LDH groups, respectively (p < 0.001), and percentage of patients with no or better leg pain (global assessment) was 74% and 65%, respectively (p = 0.008). Reoperation for recurrent LDH represented the largest independent risk for dissatisfaction; this factor and smoking represented similar risks for less improvement in leg pain.

**Conclusions:**

Repeat surgery for a recurrent LDH was performed with good probability for improvement, although not as good as for primary LDH surgery, and patients undergoing repeated surgery were less satisfied. Studies on risk factors for recurrence are warranted.

**Level of Evidence:**

Level II, therapeutic study. See Instructions for Authors for a complete description of levels of evidence.

## Introduction

Lumbar disc herniation (LDH) is one of the most common indications for lumbar spine surgery [11, 12, 19], and the lifetime incidence for disc surgery is estimated to be between 1% and 2%, although there are regional differences in and between countries [11]. The most common indications for surgery are radiating pain and neurologic symptoms resistant to nonoperative treatment, and the majority of patients who undergo surgery benefit from it [12, 19, 22]. Although removal of the herniated part of the disc is considered a standard procedure with few complications, the number of patients having a second surgical procedure within 1 or 2 years varies in the literature between 0.5% and 24% [1–3], with recurrent disc herniation being the most common cause [2, 6].

Several studies comparing outcome after surgery for LDH have not found any relevant differences between primary and revision surgery [1, 7, 16, 20]. These studies are important, although most have been of retrospective design, included limited number of patients, and used different protocols for outcome. A prospective study with a large sample size using accepted and validated patient-reported outcome measures is therefore important.

Using data from the Swedish National Spine Register, Swespine, we therefore compared patients who underwent surgery for primary LDH and patients reoperated on for recurrent LDH within 1 year after the primary operation in regard to patient-reported outcomes, including pain (VAS score), function (Oswestry Disability Index [ODI]), and quality of life (EQ-5D). We also compared patient satisfaction and global assessment with regard to leg pain between groups and investigated whether there were any factors for being less satisfied and experiencing worse global assessment with regard to leg pain.

## Patients and Methods

Swespine was initiated in 1993, and today approximately 80% of the total number of surgical procedures for LDH in Sweden is included in the register on a yearly basis [18, 19]. Approximately 90% of all spine departments register in Swespine. Preoperative questionnaire data and followup questionnaires are completed by the patients without any assistance from the surgeon. Surgical data including perioperative complications are the only information recorded by the surgeon.

The current protocol of the register, which has been validated in a test-retest situation, can reliably detect postoperative improvements between large groups of patients such as those in a registry [23, 24]. Patient identification is coded in the register, and no patient can be identified; therefore, ethical approval is not necessary according to Swedish legislation.

The followup questionnaires are sent to the patient’s home with a prepaid and addressed return envelope. Preoperative data completed by the patient include for example age, sex, and smoking habits.

The patient-reported outcomes used in this study were leg pain on a VAS scale (0–10; higher is worse), functional status using the ODI (0–100; higher is worse), and quality of life using the EQ-5D (−0.59 to 1.0; higher is better) (all validated instruments). Ordinal scale questions regarding patient satisfaction and global assessment of change in leg pain were also included. Options for satisfaction included satisfied, uncertain, and dissatisfied; options for global assessment of leg pain included pain free, much better, somewhat better, unchanged, or worse. For analysis, the answers were dichotomized as “satisfied” versus “uncertain or dissatisfied” for patient satisfaction and “completely gone or much better” versus “somewhat better, unchanged, or worse” for global assessment of leg pain. The global assessment instrument has been validated against other outcome measures [10], while the patient satisfaction instrument has not been validated.

Data were obtained for all patients reported in Swespine who underwent surgery for LDH between January 1, 2000, and April 30, 2011. Baseline data collected at the time for the primary operation (index procedure) were used for comparison at followup, meaning no new baseline data were collected before a reoperation, which is standard procedure in Swespine. We analyzed patient-reported outcomes collected 2 years after the primary operation (index procedure) for all patients. We used the 2-year followup time point after the primary operations in all patients to include at least a 1-year followup for those patients reoperated on close to 1 year after the primary operation. Since we have demonstrated that outcomes measured 1 and 2 years after LDH surgery are similar [19], we do not consider that the range of followup times distorted the results.

**Fig. 1:**
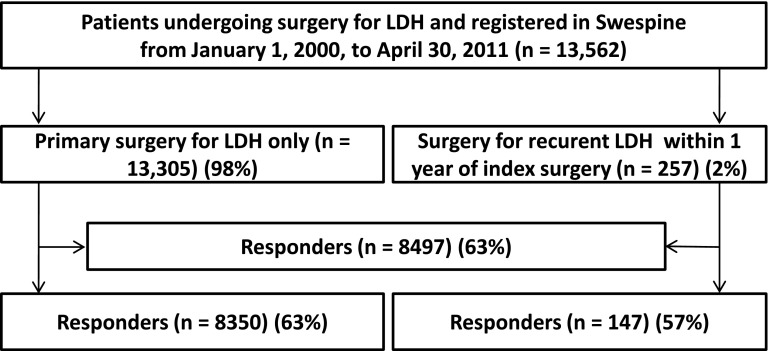
A flow diagram illustrates patient inclusion in the study.

In all, 13,562 patients who underwent surgery for an LDH due to radiating pain and neurologic symptoms resistant to nonoperative treatment were included in the analyses (Fig. 1). Of the 13,562 patients, 13,305 (98%) underwent surgery only once, for the primary LDH, and 257 (2%) underwent reoperation within the first year after the primary operation due to a recurrent LDH. The operated levels in the primary LDH group were: L5-S1 (48%), L4-L5 (41%), L3-L4 (6%), L2-L3 (1%), and other levels (4%). The operated levels in the recurrent group were L5-S1 (56%), L4-L5 (39%), L3-L4 (4%), and other levels (1%). At baseline, the groups were similar except that patients subsequently experiencing a recurrence of the herniation reported more leg pain (difference in VAS score = 5 of 100; p = 0.010) and a lower functional status (difference in ODI = 3 of 100; p = 0.026) (Table 1). However, these differences are below what is generally accepted as being clinically relevant: 15 to 20 of 100 for VAS score and 10 to 12 of 100 for ODI [9, 15].

**Table 1:** Characteristics of the two study groups at baseline

Characteristic	Primary LDH group (n = 13,305)	Recurrent LDH group (n = 257)	p value
VAS score (points)*			
Back pain	46 (29)	44 (29)	0.280
Leg pain	66 (26)	71 (22)	0.010
ODI (points)*	48 (19)	51 (18)	0.026
EQ-5D score (points)*	0.26 (0.34)	0.21 (0.32)	0.058
Age (years)*	44 (13)	43 (11)	0.207
Female (%)	44	45	0.425
Smokers (%)	23	26	0.193

* Values are expressed as mean, with SD in parentheses; VAS (0–100, higher score is worse), ODI (0–100; higher score is worse), and EQ-5D (−0.59 to 1.0; higher score is better); LDH = lumbar disc herniation; ODI = Oswestry Disability Index.

In all, 8497 of the total 13,562 patients (63%) completed the followup. There was a comparable proportion of responders in the primary LDH group (8350 of 13,305, 63%) and the recurrent LDH group (147 of 257, 57%) (p = 0.068). Loss to followup was separately analyzed in the primary and recurrent LDH groups with regard to baseline variables. In the primary LDH group, the nonresponders were more often female (p < 0.001), were slightly younger (p < 0.001), and reported a higher frequency of smoking (p < 0.001) compared to the responders; they also reported slightly less leg pain (p < 0.001) at inclusion. However, there were no differences between responders and nonresponders with regard to back pain (p = 0.195), ODI (p = 0.969), and EQ-5D (p = 0.054). In the recurrent LDH group, male sex was more common among patients lost to followup (p = 0.026). However, there were no differences between responders and nonresponders with regard to smoking (p = 0.506), age (p = 0.120), or baseline values for VAS leg pain (p = 0.620), back pain (p = 0.620), function ODI (p = 0.767), or EQ-5D (p = 0.794). We consider the statistically significant differences between responders and nonresponders to be of minor importance from a clinical perspective.

For the VAS, ODI, and EQ-5D scores, we used the difference between preoperative (primary operation) and followup values as a measure of improvement. Baseline characteristics between the groups were compared using the independent T-test for continuous data. The chi-square test was used for ordinal data (smoking, sex, number of responders). In the outcome calculations, the continuous variables were analyzed in a mulivariate fashion using the analysis of covariance test, with adjustment for sex, smoking, age at baseline, and baseline value of the analyzed variable. After dichotomization of patient satisfaction and global assessment into binary variables, adjusted logistic regression was used to express odds ratios with 95% CIs. The models were adjusted for sex, smoking, and age. In multivariate analysis, it is important to use limited numbers of relevant regressors. In large observational studies, multiple regression often restricts the number of patients possible to include in the statistical analysis. We considered age, sex, and smoking as the most important confounders in this study, although residual confounding remained.

## Results

Patients undergoing primary surgery for LDH experienced greater improvement than patients having revision surgery for a recurrent LDH within the first year after the primary operation in terms of leg and back pain (mean [95% CI] differences between groups: VAS score, 9 of 100 [4–14] and 9 of 100 [5–15], respectively), function (ODI: 6 of 100 [3–9]), and quality of life (EQ-5D: 0.09 [0.04–0.15]) (Table 2). In a multivariate analysis of the changes in scores from baseline to followup, there was greater improvement (all p < 0.001) in the primary LDH group compared with the recurrent LDH group. However, the differences in patient-reported outcomes between groups were mostly lower than what is often regarded as clinically relevant differences [9, 13, 15].

**Table 2:** Outcome improvement in the two study groups

Outcome	Recurrent LDH group			Primary LDH group			Difference between groups (95% CI)	p value
	Number of patients	Mean change from baseline to followup	95% CI	Number of patients	Mean change from baseline to followup	95% CI		
VAS (points)								
Back pain	113	12	7–17	6683	21	21–22	9 (5–15)	< 0.001
Leg pain	113	37	32–42	6719	46	45–46	9 (4–14)	< 0.001
ODI (points)	112	24	21–27	5099	31	30–31	6 (3–9)	< 0.001
EQ-5D (points)	112	0.38	0.33–0.43	5928	0.47	0.47–0.48	0.09 (0.04–0.15)	< 0.001

Calculations performed in analysis of covariance with adjustment for age, sex, smoking, and baseline value of each outcome measure; * VAS (0–100; higher score is worse), ODI (0–100; higher score is worse), and EQ-5D (−0.59 to 1.0; higher score is better); LDH = lumbar disc herniation; ODI = Oswestry Disability Index.

Patient satisfaction and global assessment of improvement in leg pain were superior in patients in the primary LDH group compared with patients in the recurrent LDH group. The proportion of patients reported to be satisfied was 79% and 58% in the primary and recurrent LDH groups, respectively (p < 0.001), and the proportion of patients with no leg pain or better leg pain was 74% and 65%, respectively (p = 0.008) (Table 3). Using the primary LDH group as reference, the adjusted odds ratio in the recurrent LDH group was 2.56 (95% CI, 1.75–3.76) (p < 0.001) for dissatisfaction and 1.48 (95% CI, 0.99–2.21) (p = 0.055) for an inferior improvement in leg pain (Table 4). In addition, in a multivariate analysis of patient satisfaction and global assessment of leg pain adjusted for recurrent LDH, male sex, smoking, and age, a reintervention for a recurrent LDH represented the largest independent risk of patient dissatisfaction. Smoking and reintervention for a recurrent LDH represented similar risks for less improvement in leg pain.

**Table 3:** Results of patient satisfaction and global assessment of leg pain

Variable	Number of patients		p value*
	Recurrent LDH group	Primary LDH group	
Satisfied^†^	83 (58%)	6441 (79%)	< 0.001
Pain free or much better^‡^	93 (65%)	6106 (74%)	0.008

* Chi-square test; ^†^options for satisfaction: satisfied, uncertain, dissatisfied; ^‡^options for global assessment of leg pain: pain free, much better, somewhat better, unchanged, worse; LDH = lumbar disc herniation.

**Table 4:** Risk of dissatisfaction and less improvement in leg pain (global assessment) with and without reoperation for a recurrent LDH*

Variable	Factor	Odds ratio	95% CI	p value
Dissatisfaction	Recurrent LDH	2.56	1.75–3.76	< 0.001
	Male sex	0.91	0.81–1.02	0.097
	Smoking	1.75	1.53–2.01	< 0.001
	Higher age	1.01	1.01–1.02	< 0.001
Less improvement in leg pain	Recurrent LDH	1.48	0.99–2.21	0.055
	Male sex	0.82	0.73–0.91	< 0.001
	Smoking	1.72	1.51–1.95	< 0.001
	Higher age	1.03	1.02–1.03	< 0.001

* Risk was analyzed in multivariate logistic regression with adjustment for sex, smoking, and age at baseline; an odds ratio of greater than 1 means increased risk of dissatisfaction/less improvement in leg pain; recurrent LDH is compared with primary LDH; LDH = lumbar disc herniation.

## Discussion

LDH is a common indication for lumbar spine surgery. The proportion of patients having a second surgical procedure within 1 or 2 years varies between 0.5% and 24% in the literature [1–3] with recurrent disc herniation being the most common cause [2, 6]. Several studies comparing outcomes after surgery for LDH have not found any relevant differences between primary and revision surgery, but these studies have some limitations, such as retrospective design and limited number of patients [1, 7, 16, 20]. A prospective study with a large sample size using patient-reported outcome measures is therefore important. Using patient-reported outcomes from Swespine, we found that 257 of 13,562 patients (2%) were operated on for a recurrent LDH within the first year after the primary operation, and 147 of these were available for analyses (57%). The patients with recurrent LDH reported slightly but significantly lower outcome scores with regard to back and leg pain (VAS scores), function (ODI), and quality of life (EQ-5D) after minimum 1-year followup. While these differences were small (and so perhaps not clinically relevant), the differences in patient satisfaction and global assessment of change in leg pain also favored the primary group and probably were large enough to be considered clinically meaningful.

This study has limitations and should be interpreted in light of these. First, in terms of followup, of the 13,562 patients included in the registry, 8497 (63%) responded after 2 years and were thus available for analyses, 8350 in the primary LDH group and 147 in the recurrent LDH group. The patient response rates was 63% and 57% in the primary and recurrent LDH groups, respectively; the difference of 6% was not significant (p = 0.068), but this should nevertheless be kept in mind. Second, there were differences in baseline variables; although the large sample size resulted in some of these being statistically significant, these differences did not reach what is considered to be relevant clinical values. Also, although there were a few baseline differences between the responders and nonresponders within the two groups and although we regard the low response rate as an issue, we still believe our results are valid.

Another limitation pertains to the effect sizes and the literature-derived minimal clinically important differences. While we found statistical differences between the study groups in some outcome measures, it is our impression from what we have reviewed in the literature that these differences are not clinically important as they did not reach reported minimally important clinical difference values, which have been reported to be about 15 to 20 of 100 for VAS [9, 15], 10 to 12 of 100 for ODI [9, 15], and 0.074 to 0.17 of 1.59 for EQ-5D [13]. It is however worth noting that this is not the most typical use of the minimally important clinical difference. Usually, this metric is used to compare differences between “before and after” for a single treatment/operation within a group and not to compare differences between groups. However, it seemed reasonable to us to use this in a cross-sectional comparison between groups.

While not a limitation per se, the question often arises about the relationship between registry data and other research designs, such as the randomized, controlled trial, in which baseline confounders may be neutralized in a way that makes it possible to draw specific conclusions about different treatment strategies. We believe these designs are complementary, in that randomized controlled trials offer strong internal validity, but registry data may be superior in terms of external validity (generalizability) [5, 14]. In a study published in 2000, Benson and Hartz [4] “found little evidence that estimates of treatment effects in observational studies reported after 1984 are either consistently larger than or qualitatively different from those obtained in randomized, controlled trials.” Along a similar line, it is important to consider whether patients lost to followup in a registry differ from those lost to followup in clinical trials, as is often the case. In a study from Norway, patient-reported outcomes were documented in a spine registry after 1 year, and the results were compared with the outcomes among patients not reporting in the registry (22%), without finding any differences in baseline characteristics or risk factors [17]. It can therefore be argued that patients not reporting in a registry may differ from those patients not reporting in a randomized, controlled trial and that it cannot be taken for granted that those not reporting in a registry are those with less successful outcomes [17].

Our results contrast somewhat from what was reported by Ahsan et al. [1]. They included 398 patients surgically treated for primary LDH and 18 patients subsequently operated on for a recurrent LDH. One year after surgery, no significant difference in radicular pain (VAS) or disability status (ODI) was found; 85% in the primary LDH group and 78% in the recurrent LDH group reported excellent or good outcomes. That study however did not adjust for any baseline differences. Another study found improvements similar to ours in leg and back pain and function (ODI) when comparing surgical treatment for primary and recurrent LDH [16]. The study design was retrospective and included 30 patients during a period of 13 years. Two senior authors performed all the reoperations with or without fusion, and the authors concluded that larger multicenter studies are warranted. A study performed at two hospitals in different countries compared 24 patients undergoing surgery for recurrent LDH and 50 patients for primary LDH [7]. A 100-point grading system was used to assess the overall clinical outcome, including severity of pain, functional status, patient satisfaction, and the result of physical examination. According to this grading system, 81% in the recurrent LDH group and 86% in the primary LDH group had excellent or good results (p > 0.05). Suk et al. [20] included 26 patients treated surgically for ipsilateral or contralateral recurrent disc herniation on the same level as the primary surgery. The study was retrospective and no differences in improvement between groups were found.

Compared with our study, these earlier studies had different criteria for inclusion, followup periods, approaches, surgical treatments, outcome measurements, sample sizes, and numbers of surgeons, clinics, and patients. This may help explain the differences between their results and ours, where we found that patients on a group level had better outcome if they were not reoperated for a recurrent LDH within 1 year after the primary operation.

Interestingly, although we believe that the differences in reported outcomes with regard to leg pain (VAS), function (ODI), and quality of life (EQ-5D) are close to or below the reported minimally clinically important differences for these scores, patients were more likely to not be satisfied (odds ratio = 2.56) and have less improvement in global assessment of leg pain (odds ratio = 1.48) if they had undergone an operation for a recurrent LDH. To experience a reoperation is probably psychologically negative, which could explain the difference between patient satisfaction and 1-year patient-reported outcomes in regard to pain, function, and quality of life. While patient satisfaction is considered to be a relevant measure in patient-centered care, we find it interesting that the correlation between satisfaction and other outcome measures such as VAS, ODI, and EQ-5D has been demonstrated to be poor [8, 21], and it is important to note that patient satisfaction was not measured using a validated tool in this study. In contrast, patient global assessment of leg pain before and after surgery has been reported to be a valid outcome tool for the overall effect of surgical spine care [10]. In all, we consider both patient satisfaction and global assessment as important outcome measures, but they may measure different aspects of spine surgery compared with patient-reported outcomes in terms of pain, function, and quality of life [8, 21].

We found that repeat surgery for a recurrent LDH can be performed with a high likelihood of clinical improvement, although not as high as for primary LDH surgery, and patients undergoing reoperation were less satisfied with their result. Future studies on risk factors for recurrence are warranted. We believe that registers measuring patient-reported outcomes should be using an internationally agreed on “core data set” with relevant followup periods. Such a set, basically built on patient-reported outcome measures, has been launched recently (November 2013) for low-back pain with the International Consortium of Health Outcome Measurements as coordinator (www.ichom.org) and with current participation of approximately 20 countries on a global scale. Results from these comparisons will hopefully facilitate benchmarking and increase our possibilities to improve spine surgery faster to the benefit of our patients.
